# Glycemic Outcomes After Switching from SGLT2 Inhibitors to DPP-4 Inhibitors in Type 2 Diabetes, with a Comparison of Teneligliptin and Other Agents: A Retrospective Cohort Study

**DOI:** 10.3390/medicina62081511

**Published:** 2026-08-06

**Authors:** Joung Youl Lim, Minchul Song, Yea Eun Kang, Ju Hee Lee, Hyun Jin Kim, Kyong Hye Joung, Bon Jeong Ku

**Affiliations:** 1 Department of Internal Medicine, Chungnam National University College of Medicine, Daejeon 35015, Republic of Korea; adslzang@cnuh.co.kr (J.Y.L.); mcsong1226@cnuh.co.kr (M.S.); yeeuni220@cnuh.co.kr (Y.E.K.); serenaju@cnuh.co.kr (J.H.L.); kimhj43@cnuh.co.kr (H.J.K.); 2 Department of Endocrinology and Metabolism, Chungnam National University Hospital, Daejeon 35015, Republic of Korea; 3 Department of Endocrinology and Metabolism, Chungnam National University Sejong Hospital, Sejong 30099, Republic of Korea

**Keywords:** type 2 diabetes mellitus, SGLT2 inhibitors, DPP-4 inhibitors, teneligliptin, glycated hemoglobin, drug switching, glycemic control

## Abstract

*Background and Objectives*: Sodium–glucose cotransporter-2 (SGLT2) inhibitors provide cardio-renal protection in type 2 diabetes, but tolerability-related discontinuation is common, creating a need for effective replacement therapy. Dipeptidyl peptidase-4 (DPP-4) inhibitors are widely used as follow-on agents, yet the clinical course after such a switch has not been systematically characterized, and whether individual DPP-4 inhibitors differ in efficacy in this setting is unknown. *Materials and Methods*: We conducted a single-center retrospective cohort study at Chungnam National University Hospital (Daejeon, Republic of Korea) between January 2013 and January 2025, including 117 adults with type 2 diabetes who switched from an SGLT2 inhibitor to a DPP-4 inhibitor and met pre-specified criteria for medication stability and follow-up. The primary outcome was the change in glycated hemoglobin (HbA1c) from baseline to 3 months; secondary outcomes were changes in body weight, body mass index, blood pressure, and renal parameters. A pre-specified subgroup analysis compared teneligliptin (*n* = 60) with other DPP-4 inhibitors as a class (*n* = 57). *Results*: In the overall cohort, body weight rose by 0.7 kg and systolic blood pressure by 4.3 mmHg at 3 months (both *p* < 0.05), whereas HbA1c was unchanged (*p* = 0.157). In the subgroup analysis, HbA1c fell significantly with teneligliptin (−0.36%; 95% confidence interval, −0.62 to −0.10; *p* = 0.009) but not with other DPP-4 inhibitors (+0.13%). The between-group difference was −0.49% (95% confidence interval, −0.83 to −0.15; *p* = 0.005) and persisted after adjustment for baseline HbA1c and estimated glomerular filtration rate (*p* = 0.007). *Conclusions*: Switching preserved overall glycemic control at 3 months but produced modest, anticipated increases in body weight and blood pressure. Teneligliptin was associated with a greater HbA1c reduction than the pooled group of other DPP-4 inhibitors; this association persisted after adjustment for the two available baseline covariates but could not be adjusted for diabetes duration, medication adherence, diabetic complications, or other unmeasured factors. Because confounding by indication and other residual confounding cannot be excluded in this short-term, single-center analysis, this finding is hypothesis-generating only and requires confirmation in adequately powered prospective head-to-head trials. Importantly, this study evaluated only the glucose-lowering effect of the switch; because the cardio-renal protection of SGLT2 inhibition is not reproduced by DPP-4 inhibitors, a DPP-4 inhibitor should be regarded as an unavoidable substitute when an SGLT2 inhibitor cannot be maintained rather than a therapeutically equivalent replacement, and preserved HbA1c at 3 months does not establish clinical equivalence between the two strategies.

## 1. Introduction

Sodium–glucose cotransporter-2 (SGLT2) inhibitors are now established as a cornerstone of therapy for type 2 diabetes, providing cardio-renal protection that extends well beyond glucose lowering [1,2,3,4,5]. Reflecting this evidence, current American Diabetes Association guidelines position cardio-renal protection alongside glycemic control as a primary therapeutic goal [6]. In real-world practice, however, sustained use of SGLT2 inhibitors is often not achieved. Tolerability-related adverse events—such as recurrent genital and urinary tract infections, polyuria and nocturia, volume depletion, excessive weight loss, and, rarely, euglycemic diabetic ketoacidosis—drive substantial discontinuation: a 3-year post-marketing surveillance study reported a discontinuation rate of approximately 10% [7], and a real-world heart-failure cohort reported up to 20% discontinuation by 5 years [8]. Because the cardio-renal benefits of these agents accrue only when therapy is sustained, tolerability-related discontinuation directly erodes their population-level effectiveness. There is therefore an unmet clinical need to define an effective treatment strategy for patients in whom SGLT2 inhibitors are indicated but cannot be maintained.

In this context, dipeptidyl peptidase-4 (DPP-4) inhibitors represent a clinically attractive alternative. They are characterized by excellent tolerability, a low incidence of adverse events, and intermediate glucose-lowering efficacy that improves postprandial glucose without inducing hypoglycemia [9]. In a comparative cohort study of seven antidiabetic drug classes, DPP-4 inhibitors achieved the highest treatment persistence among all classes evaluated, including biguanides, sulfonylureas, and SGLT2 inhibitors [10]. These features make DPP-4 inhibitors a logical replacement option when SGLT2 inhibitors must be discontinued for tolerability reasons.

Despite often being viewed as a homogeneous class in clinical practice, DPP-4 inhibitors are pharmacologically heterogeneous. Individual agents differ in molecular structure, binding mode to the DPP-4 enzyme, half-life, and excretion pathway, and these differences may translate into clinically relevant variations in potency and pleiotropic effects [11]. Although most DPP-4 inhibitors have shown neutral results in large-scale cardiovascular outcome trials, head-to-head studies have observed differences in glycemic control and low-density lipoprotein cholesterol reduction among agents, and preclinical experiments have identified mechanistic distinctions such as differential β-cell protection [12,13,14,15]. Drug-specific signals, such as the heart failure risk associated with some agents but not others, further argue against treating the class as uniform [16]. Teneligliptin, in particular, has dual renal and hepatic excretion pathways that allow once-daily dosing without renal dose adjustment [17] and has demonstrated intrinsic antioxidant activity in preclinical studies, although direct head-to-head clinical evidence of superior efficacy over other class members is lacking [18].

To our knowledge, no previous study has systematically examined the clinical outcomes of patients who switch from an SGLT2 inhibitor to a DPP-4 inhibitor, nor has any study compared individual DPP-4 inhibitors in this specific clinical context. Whether the within-class heterogeneity described above is clinically apparent in this scenario therefore remains unknown. We aimed to (*i*) characterize the clinical changes that occur after switching from an SGLT2 inhibitor to a DPP-4 inhibitor, regardless of the reason for discontinuation, and (*ii*) compare glycemic outcomes between teneligliptin and other DPP-4 inhibitors as a class. Two pre-specified hypotheses were tested: (*a*) that overall glycemic control would be maintained at 3 months after the switch, with a partial return of the metabolic and hemodynamic effects (body weight, blood pressure) attributable to SGLT2 inhibition; and (*b*) that the magnitude of HbA1c response would differ between teneligliptin and the aggregated comparator group. Teneligliptin was chosen as the reference comparator because it is one of the most frequently prescribed DPP-4 inhibitors in the Republic of Korea according to the UBIST nationwide pharmaceutical market research database [19] and was the most frequently prescribed agent at our institution during the study period, providing a sufficient sample size for comparison.

## 2. Materials and Methods

### 2.1. Study Design and Population

This was a single-center retrospective cohort study of adults with type 2 diabetes who switched from an SGLT2 inhibitor to a DPP-4 inhibitor at Chungnam National University Hospital, a tertiary referral center in Daejeon, Republic of Korea, between 1 January 2013 and 31 January 2025. All eligible patients during this period were identified from the hospital electronic medical record system, and consecutive cases meeting eligibility criteria were included to minimize selection bias. Data were accessed for research purposes from 1 February 2025. The study protocol was approved by the Institutional Review Board of Chungnam National University Hospital (IRB No. 2024-08-037), which waived the requirement for informed consent given the retrospective design and the use of anonymized data. The study was conducted in accordance with the Declaration of Helsinki and is reported in accordance with the STROBE statement for observational studies.

Inclusion criteria were as follows: (*i*) treatment with an SGLT2 inhibitor for at least 3 months without changes in agent or dose prior to switching; (*ii*) maintenance of a DPP-4 inhibitor for at least 3 months without changes in agent or dose after switching; and (*iii*) no change in the type or dose of other oral antidiabetic agents and no insulin use at the time of switching. Patients with insufficient pre- and post-switch HbA1c data or with concomitant changes in other glucose-lowering medications were excluded.

Clinical data were collected at the time of switching (baseline) and at 3 months after switching. The two-group comparison—teneligliptin versus all other DPP-4 inhibitors as a class—was specified in the original study protocol approved by the Institutional Review Board (IRB No. 2024-08-037) before any analysis was performed; the rationale was that teneligliptin was the most frequently prescribed agent at our institution and one of the most frequently prescribed DPP-4 inhibitors in the Republic of Korea, which provided sufficient sample size for a meaningful comparison and prevented post hoc selection of comparator agents. Changes in clinical parameters between baseline and 3 months were then compared between the two pre-specified groups. Baseline values were defined as the measurements obtained closest to, and no more than 3 months before, the switch date, and 3-month values as the measurements obtained closest to 90 days after switching within a window of 1 to 5 months; when more than one measurement was available within a window, the value closest to the nominal time point was used, and measurements outside these windows were not carried forward.

### 2.2. Clinical and Laboratory Assessments

Clinical parameters comprised age, sex, body weight, body mass index, and systolic and diastolic blood pressure. Height and weight were measured by trained nurses in the outpatient clinic. Blood pressure was measured in the right upper arm using an unattended automated office blood pressure device after at least 5 min of seated rest in the waiting area.

Laboratory parameters comprised aspartate aminotransferase, alanine aminotransferase, blood urea nitrogen, serum creatinine, estimated glomerular filtration rate (eGFR), urinary albumin-to-creatinine ratio, and glycated hemoglobin (HbA1c). All measurements were performed using an automated clinical chemistry analyzer (TBA-FX8, Canon Medical Systems, Tokyo, Japan). HbA1c was measured by high-performance liquid chromatography aligned with the National Glycohemoglobin Standardization Program and standardized to the Diabetes Control and Complications Trial reference. eGFR was calculated using the Chronic Kidney Disease Epidemiology Collaboration equation. Reasons for switching were extracted from the electronic medical records. For terminology, switching attributable to a documented tolerability-related adverse event (e.g., genital or urinary tract infection, osmotic-diuresis symptoms, volume depletion, or excessive weight loss) was distinguished from switching recorded for poor glycemic control, insurance reasons, or with no documented reason; these categories are not equivalent to drug intolerance. “Poor glycemic control” as a documented reason reflected the treating physician’s judgment of an inadequate HbA1c response, generally an HbA1c above the guideline-based target of 7.0%. A single uniform target was not applied and individualized targets were not systematically recorded, and the option of intensifying therapy without withdrawing the SGLT2 inhibitor was left to the treating physician and was not captured; these documented reasons should therefore be interpreted with caution. For patients with an established heart-failure, chronic-kidney-disease, or atherosclerotic-cardiovascular-disease indication, current guidelines would favor maintaining the SGLT2 inhibitor despite suboptimal glycemic control, but such organ-protective indications were not reliably documented during the 2013–2025 study period (see Limitations).

### 2.3. Statistical Analysis

All statistical analyses were performed using SPSS version 26.0 (IBM Corp., Armonk, NY, USA). Continuous variables are presented as mean ± standard deviation, and categorical variables as number (percentage). Within-group changes from baseline to 3 months were assessed using paired *t*-tests. Between-group differences in baseline values and in the magnitude of change were assessed using independent-samples *t*-tests, and categorical variables were compared using the chi-square or Fisher’s exact test as appropriate. To assess baseline equivalence between the teneligliptin and other DPP-4 inhibitors groups—a key concern in any non-randomized comparison—we examined both conventional *p*-values and absolute differences for all candidate confounders. Pearson correlation analysis was used to evaluate associations between continuous variables. The primary effect estimate for the pre-specified between-group comparison of HbA1c change was the unadjusted mean difference; the principal sensitivity analysis was a multivariable linear regression adjusted for baseline HbA1c and baseline eGFR, the two pre-specified clinical confounders. Baseline HbA1c was selected because of the well-known regression-to-the-mean effect on HbA1c reduction, and baseline eGFR was selected because renal function influences both DPP-4 inhibitor pharmacokinetics and prescriber choice between agents. The cohort size was determined by the patients available during the study period rather than by an *a priori* power calculation; given the observed pooled standard deviation of HbA1c change (0.96%) and the achieved sample size of 117, the available precision was sufficient to detect a between-group HbA1c difference of approximately 0.4 percentage points at α = 0.05 with 80% power. This estimate assumes a simple unadjusted two-group comparison and does not account for covariate adjustment in the multivariable models or for the multiple secondary comparisons performed; the effective precision of the adjusted analyses is therefore lower than this figure implies. Two-tailed *p*-values < 0.05 were considered statistically significant. Because secondary outcomes were considered exploratory and outnumbered the primary outcome, no correction for multiple testing was applied; results for outcomes other than HbA1c should therefore be interpreted as hypothesis-generating. Because the urinary albumin-to-creatinine ratio was markedly right-skewed, it was summarized as the median (interquartile range) and analyzed with non-parametric tests (Wilcoxon signed-rank test for within-group change and Mann–Whitney U test for between-group comparison). The number of available observations is reported for every clinical and laboratory variable, because missingness differed across variables and was greatest for the urinary albumin-to-creatinine ratio (available in 63 of 117 patients at baseline). For the principal between-group comparisons, the change from baseline is presented as the mean change with its 95% confidence interval rather than as within-group *p*-values alone.

## 3. Results

### 3.1. Baseline Characteristics and Reasons for Switching

**Figure 1:**
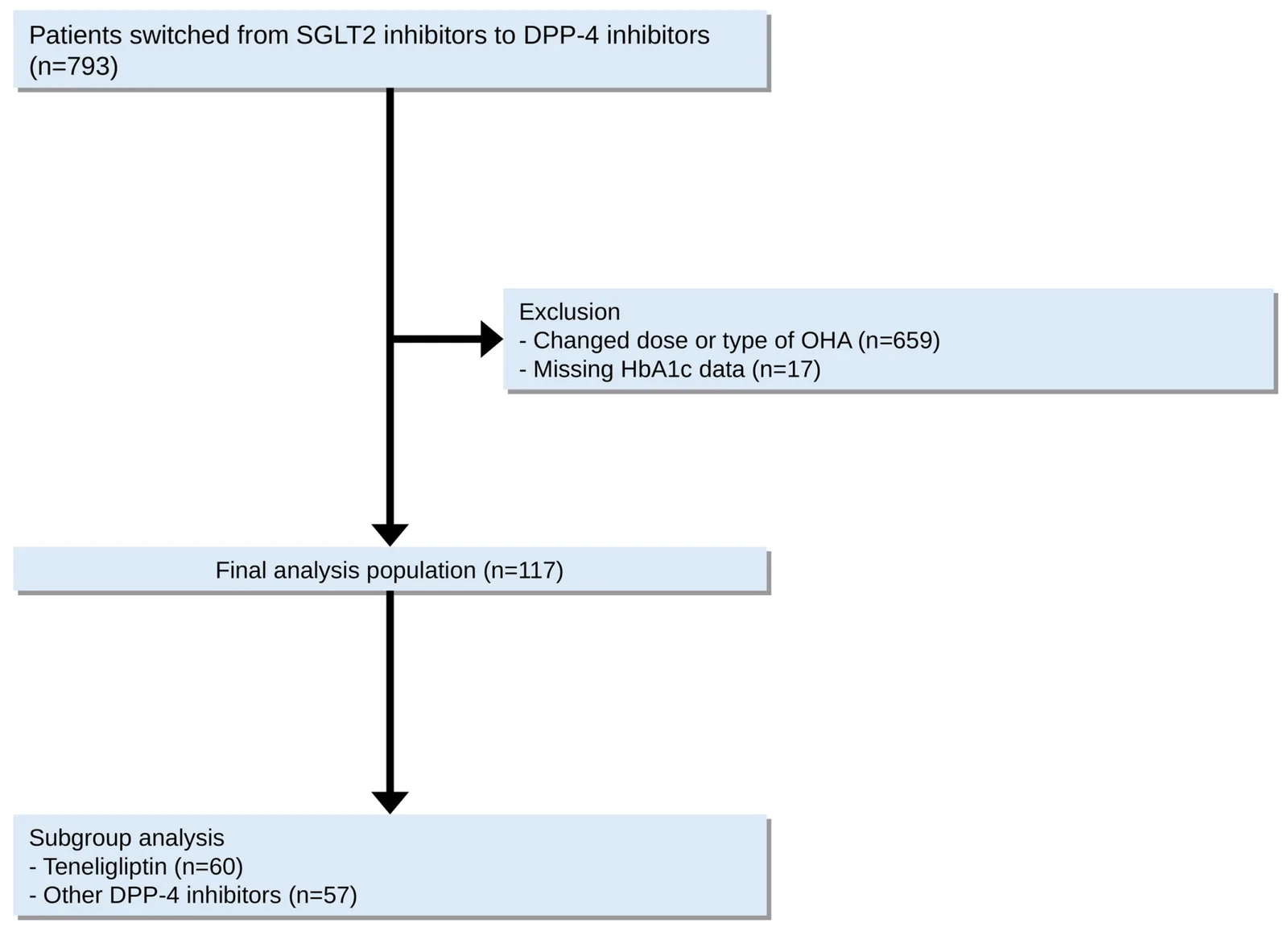
Flowchart of study population selection. Out of 793 patients screened for switching from SGLT2 inhibitors to DPP-4 inhibitors, those with changes in other oral hypoglycemic agents or missing HbA1c data were excluded. A final total of 117 patients were included and stratified into the teneligliptin group (*n* = 60) and other DPP-4 inhibitors group (*n* = 57). SGLT2, sodium-glucose cotransporter-2; DPP-4, dipeptidyl peptidase-4; HbA1c, glycated hemoglobin; OHA, oral hypoglycemic agent.

Of 793 patients identified as having switched from an SGLT2 inhibitor to a DPP-4 inhibitor during the study period, 117 met all inclusion criteria and were included in the final analysis (Figure 1). Reasons for exclusion are shown in Figure 1. The mean (standard deviation) age of the cohort was 60.5 (11.1) years, 54.7% were men, and the baseline body mass index was 25.5 (3.2) kg/m^2^ (Table 1). The most commonly used pre-switch SGLT2 inhibitors were dapagliflozin (47.9%) and empagliflozin (46.2%); enavogliflozin (2.6%) and ipragliflozin (3.4%) accounted for the remainder. The most frequently prescribed post-switch DPP-4 inhibitors were teneligliptin (51.3%), gemigliptin (19.7%), and sitagliptin (11.1%); the remaining patients (17.9%) received vildagliptin, alogliptin, linagliptin, anagliptin, or evogliptin. A documented reason for switching was identified in the electronic medical record for 84 patients (71.8%); the most common were poor glycemic control (16.2%) and genital mycotic infection (15.4%) (Table 2). Additional tolerability-related reasons included weight loss (12.0%) and nocturia (6.8%). For the remaining 33 patients (28.2%), no specific reason was documented in the medical record at the time of switching; this category was retained for transparency but excluded from the regression analysis of reasons. In a univariable linear regression analysis, none of the documented reasons for switching were significantly associated with the change in HbA1c after switching (Table S1). The number of available observations for each variable is reported in Table 1, Table 3, Table 4, Table 5, Table 6 and Table 7; the urinary albumin-to-creatinine ratio was the most incomplete variable, available in 63 patients (33 in the teneligliptin group and 30 in the other DPP-4 inhibitors group) at baseline.

### 3.2. Overall Clinical Changes After Switching

In the overall cohort, the principal post-switch changes were modest increases in body weight and blood pressure. Mean body mass index increased from 25.5 (3.2) to 25.9 (3.3) kg/m^2^ (mean change, +0.4 kg/m^2^; *p* < 0.001), and body weight increased from 67.5 (9.4) to 68.2 (9.8) kg (mean change, +0.7 kg; *p* < 0.001) (Table 3). Systolic and diastolic blood pressure increased by a mean of 4.3 mmHg (*p* = 0.005) and 1.9 mmHg (*p* = 0.042), respectively. No significant changes were observed in aspartate aminotransferase, alanine aminotransferase, blood urea nitrogen, serum creatinine, eGFR, urinary albumin-to-creatinine ratio, or HbA1c. Mean HbA1c was 7.7% (1.4) at baseline and 7.6% (1.5) at 3 months (*p* = 0.157). In a multivariable linear regression of HbA1c change, lower baseline eGFR was independently associated with a greater HbA1c reduction (β = 0.01 per mL/min/1.73 m^2^; 95% confidence interval, 0.00 to 0.02; *p* = 0.009; Table S2). Because the urinary albumin-to-creatinine ratio was markedly right-skewed, within- and between-group analyses of this variable were repeated non-parametrically and are reported as the median (interquartile range); the non-parametric analyses did not alter the conclusion of no significant renal change after switching.

### 3.3. Subgroup Analysis: Teneligliptin Versus Other DPP-4 Inhibitors

Of the 117 patients, 60 (51.3%) were switched to teneligliptin and 57 (48.7%) to another DPP-4 inhibitor. Baseline characteristics—including age, sex, body mass index, blood pressure, renal function, and the distribution of prior SGLT2 inhibitor agents—were comparable between the two groups (all *p* ≥ 0.05), as were baseline laboratory parameters (aspartate aminotransferase, alanine aminotransferase, blood urea nitrogen, serum creatinine, eGFR, urinary albumin-to-creatinine ratio, and HbA1c) (Table 4). The most notable baseline differences, both reaching only borderline statistical significance, were in diastolic blood pressure (79.2 vs. 75.3 mmHg; *p* = 0.057), which is unlikely to materially influence the primary glycemic outcome. Baseline HbA1c was also marginally higher in the teneligliptin group than in the other DPP-4 inhibitors group (7.9% vs. 7.5%; *p* = 0.050); because a higher baseline value could favor a larger reduction through regression to the mean, baseline HbA1c was included as a pre-specified covariate in the multivariable model, and the between-group difference in HbA1c change remained significant after this adjustment.

Within-group changes differed between the two groups. The other DPP-4 inhibitors group showed significant within-group increases in body weight, body mass index, systolic blood pressure, and diastolic blood pressure at 3 months, whereas the teneligliptin group showed significant within-group increases in body weight and body mass index. The post-switch increase in systolic blood pressure tended to be smaller in the teneligliptin group than in the other DPP-4 inhibitors group (between-group *p* = 0.050). Within-group results are shown in Table 5 for the teneligliptin group and Table 6 for the other DPP-4 inhibitors group, and between-group comparisons of the change from baseline are shown in Table 7.

**Figure 2:**
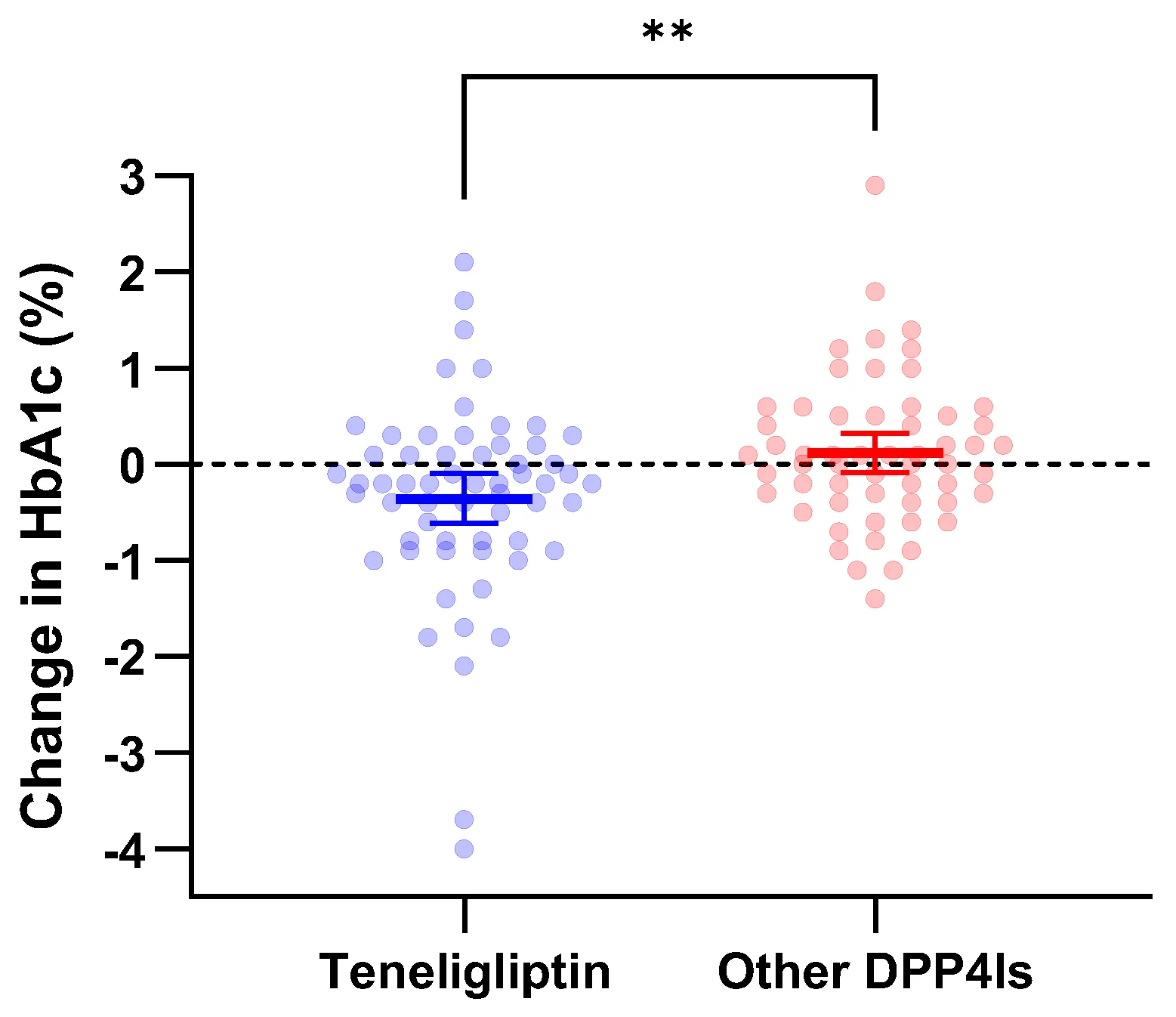
Change in HbA1c from baseline to 3 months in the teneligliptin and other DPP-4 inhibitors groups. Each dot represents an individual patient; the horizontal line and error bars indicate the group mean and its 95% confidence interval. ** indicates *p* < 0.01 for the between-group difference in HbA1c change. DPP-4is, dipeptidyl peptidase-4 inhibitors; HbA1c, glycated hemoglobin.

For the primary outcome, mean HbA1c decreased significantly in the teneligliptin group (mean change, −0.36% [standard deviation, 1.02]; −4 mmol/mol [standard deviation, 11]; *p* = 0.009), whereas it did not change significantly in the other DPP-4 inhibitors group (mean change, +0.13% [standard deviation, 0.76]; +1 mmol/mol [standard deviation, 8]; *p* = 0.236). The mean between-group difference in HbA1c change was −0.49% (95% confidence interval, −0.83 to −0.15; −5 mmol/mol [95% confidence interval, −9 to −2]; *p* = 0.005) in favor of teneligliptin (Figure 2), and remained significant after adjustment for baseline HbA1c and baseline eGFR in multivariable linear regression (adjusted β = −0.57; 95% confidence interval, −0.97 to −0.16; *p* = 0.007; Table S2). A non-significant trend toward a smaller post-switch increase in urinary albumin-to-creatinine ratio was observed in the teneligliptin group compared with the other DPP-4 inhibitors group (median change, −4.3 mg/g [range, −27.9 to 3.1] in the teneligliptin group vs. +2.5 mg/g [range, −10.8 to 21.9] in the other DPP-4 inhibitors group; Mann–Whitney U test, *p* = 0.090; Table 7). Although this comparison was exploratory and did not reach statistical significance, the direction is consistent with previously reported antioxidant and renal-protective signals for teneligliptin [18,20] and warrants further evaluation in adequately powered studies. The between-group differences in the change from baseline are reported as the mean change (95% confidence interval) in each group in Table 7; the post-switch rise in systolic blood pressure was smaller with teneligliptin (2.3 mmHg [−2.7 to 7.3]) than with other DPP-4 inhibitors (10.1 mmHg [3.8 to 16.3]; *p* = 0.050), as was the change in body weight (0.6 kg [0.1 to 1.1] vs. 1.2 kg [0.4 to 2.1]; *p* = 0.217).

## 4. Discussion

In this single-center retrospective cohort of 117 adults with type 2 diabetes, switching from an SGLT2 inhibitor to a DPP-4 inhibitor produced three principal findings. First, overall glycemic control was preserved at 3 months, with no significant change in mean HbA1c. Second, the switch was accompanied by modest but statistically significant increases in body weight (+0.7 kg) and systolic blood pressure (+4.3 mmHg), consistent with loss of the metabolic and hemodynamic effects characteristic of SGLT2 inhibition. Third, in a pre-specified subgroup analysis, patients switched to teneligliptin had a 0.49 percentage-point greater reduction in HbA1c than those switched to other DPP-4 inhibitors as a class (95% confidence interval, 0.15 to 0.83), and this between-group difference remained significant after adjustment for baseline HbA1c and eGFR. The magnitude of this difference is not trivial: it approximates one-half of the average HbA1c reduction achieved by DPP-4 inhibitors as monotherapy in randomized trials (typically 0.5–1.0%) and exceeds the 0.3–0.4 percentage-point threshold commonly cited as the minimal clinically important difference for HbA1c in type 2 diabetes [11,21]. This clinical-significance framing, however, presupposes that the observed difference reflects a genuine pharmacological effect; given the non-randomized design and the marginally higher baseline HbA1c in the teneligliptin group (7.9% vs. 7.5%; *p* = 0.050), part or all of the difference could instead reflect regression to the mean and confounding by indication rather than a true between-agent effect. To our knowledge, this is the first real-world study to characterize the clinical course of switching from SGLT2 inhibitors to DPP-4 inhibitors and to suggest that the magnitude of glycemic response in this scenario may differ between individual DPP-4 inhibitors. Given the observational design, however, these findings should be regarded as hypothesis-generating rather than confirmatory. It must be emphasized that preservation of mean HbA1c reflects short-term glycemic substitution only and does not establish clinical or therapeutic equivalence between the two strategies; a DPP-4 inhibitor should be viewed as an unavoidable option when an SGLT2 inhibitor cannot be maintained, not as an equivalent replacement. Moreover, because individualized HbA1c targets, baseline target attainment, frailty, functional status, and hypoglycemia were not captured, the clinical value of an approximately 0.5 percentage-point between-group HbA1c difference cannot be firmly established and may be limited in some patients.

Tolerability-related discontinuation of SGLT2 inhibitors is well documented and clinically substantial. In the STELLA-LONG TERM 3-year post-marketing surveillance study of ipragliflozin in Japan, approximately 10% of 8763 patients (*n* = 830) discontinued treatment because of adverse events [7]. Even in the heart failure setting—where SGLT2 inhibitors clearly improve symptoms and outcomes—real-world discontinuation rates reach 7.5% at 1 year and 20% at 5 years [8]. The leading reasons for discontinuation are genitourinary infections, osmotic diuresis-related symptoms (urinary frequency, nocturia), volume depletion, and weight loss; in the STELLA-LONG TERM cohort, 61%, 54%, and 36% of patients with genital infections, urinary tract infections, and volume depletion-related events, respectively, discontinued the drug as a result [7]. The prescription rate of SGLT2 inhibitors is also lower in women, who carry a higher risk of genitourinary infections [8]. Identifying an effective replacement therapy is therefore clinically important. According to the 2021 Korean Diabetes Fact Sheet, DPP-4 inhibitors are included in over 30% of antidiabetic prescriptions in Korea, second only to metformin (81.7%) [22], reflecting their wide acceptance as a follow-on agent in routine practice. Despite this widespread substitution pattern, however, the clinical course following such a switch has not been characterized in any systematic real-world study. Our findings address this gap by quantifying both the glycemic preservation and the metabolic–hemodynamic costs of the switch, and by providing the first comparative data on within-class differences in glycemic response.

Our finding that mean HbA1c was preserved at 3 months after the switch is clinically reassuring and suggests that DPP-4 inhibitors can substitute for the glucose-lowering effect of SGLT2 inhibitors over the short term in selected patients. It is critical, however, to distinguish glycemic substitution from the cardio-renal benefits of SGLT2 inhibition: DPP-4 inhibitors as a class have demonstrated cardiovascular and renal neutrality in large-scale outcome trials [23], whereas SGLT2 inhibitors confer well-established reductions in major adverse cardiovascular events, hospitalization for heart failure, and progression of chronic kidney disease [3,4,5]. The accompanying post-switch increases in body weight and blood pressure, although modest in absolute terms, are consistent with loss of the natriuretic, diuretic, and caloric-loss effects of SGLT2 inhibition and serve as a quantitative reminder that the cardio-renal pharmacology cannot be replicated by DPP-4 inhibitors. These changes therefore warrant particular attention in patients with established cardiovascular or renal disease for whom SGLT2-inhibitor-specific benefits were the primary indication. Within this overall pattern, the within-class heterogeneity we observed—a greater HbA1c reduction with teneligliptin than with other DPP-4 inhibitors—persisted after adjustment for baseline confounders, and lower baseline eGFR was independently associated with greater HbA1c reduction; these subgroup observations do not alter the broader cardio-renal trade-off that the switch entails. The concurrent increases in body weight and blood pressure indicate that the switch was neither metabolically nor hemodynamically neutral—a consideration of particular importance in patients for whom the original indication for SGLT2 inhibition was cardiovascular, heart-failure, or renal protection rather than glycemic control. Most patients in our cohort are presumed to have received an SGLT2 inhibitor primarily for glycemic control; however, because the study spanned 2013–2025—a period during which prevailing guidelines did not emphasize heart-failure, chronic-kidney-disease, and atherosclerotic-cardiovascular-disease indications as strongly as current (2026) recommendations—the original indication was often not explicitly documented and could not be reliably ascertained (see Limitations).

DPP-4 inhibitors lower HbA1c by inhibiting the degradation of endogenous incretins and thereby elevating circulating concentrations of glucose-dependent insulinotropic polypeptide and glucagon-like peptide-1 [11,24]. Although individual agents differ in molecular structure, binding mode, and enzyme selectivity, head-to-head comparisons in patients with stable disease have generally shown only modest differences in glycemic control or in incretin elevation [21], and large-scale cardiovascular outcome trials have demonstrated cardiovascular and renal neutrality rather than superiority across the class [23]. As a consequence, DPP-4 inhibitors are often treated as interchangeable in clinical practice, and comparative-effectiveness research within the class has been limited.

Emerging evidence, however, increasingly challenges this perception of uniformity. Randomized trials have documented superior glucose-lowering with specific agents in selected populations [14], observational studies have reported additional HbA1c reductions when patients are switched between agents within the class [25], drug-specific safety signals such as heart failure risk have been observed for some but not all agents [26], and preclinical work has shown differential endothelial protection and antioxidant activity [27]. Network meta-analyses comparing individual DPP-4 inhibitors suggest small numerical differences in HbA1c-lowering efficacy, although confidence intervals overlap and the differences are typically considered modest [11,21]. Our findings extend this body of evidence by suggesting that, in the specific context of switching from an SGLT2 inhibitor, between-agent differences within the DPP-4 inhibitor class may become more clinically apparent, and they argue for renewed comparative-effectiveness research and attention to optimal medication sequencing rather than treating the class as homogeneous. We emphasize that these observations do not establish the superiority of teneligliptin over other individual DPP-4 inhibitors and should be interpreted strictly as hypothesis-generating.

Several pharmacological features of teneligliptin may help explain its apparent advantage in this scenario, although none can be conclusively linked to the observed difference from the present data. Its distinctive “J-shaped” five-ring scaffold forms an “anchor-lock” interaction with the S2 extensive subsite of DPP-4, producing strong and prolonged enzyme inhibition; the resulting slow dissociation rate and long effective half-life support 24 h glycemic control with once-daily dosing [28]. In addition, teneligliptin exhibits intrinsic antioxidant activity in vitro and in vivo [18], and small clinical studies have reported improvements in endothelial function and vascular oxidative stress relative to sitagliptin [20,29]. Its dual renal–hepatic excretion pathway, which obviates renal dose adjustment [17], is particularly relevant in our cohort: lower baseline eGFR was independently associated with greater HbA1c reduction in the multivariable model (β = 0.01 per mL/min/1.73 m^2^; *p* = 0.009), and the cohort had relatively preserved but heterogeneous renal function (mean eGFR 89.3 mL/min/1.73 m^2^; standard deviation 22.6). It is plausible that uniform dosing without renal-function-based titration provided a modest but consistent exposure advantage in patients with mild renal impairment, although this remains speculative. Importantly, the mechanistic studies cited above have not directly demonstrated that teneligliptin achieves superior glucose-lowering compared with other DPP-4 inhibitors in head-to-head trials; the few existing comparisons report similar HbA1c reductions [30,31]. An alternative—and perhaps equally plausible—interpretation is therefore that residual confounding by prescriber preference or unmeasured baseline factors contributed to the observed between-group difference. The implications of this possibility are considered in detail in the next subsection. These pharmacological considerations are mechanistic and hypothesis-generating; none has been shown to translate into superior glucose-lowering in head-to-head trials, and this section should be read in that light. We also note that the study received an unrestricted research grant from the Korean distributor of teneligliptin (see Funding and Conflicts of Interest), and the drug-specific interpretations above are therefore presented with corresponding caution.

### Strengths and Limitations

This study has several strengths. First, it addresses a clinically important but under-investigated scenario—the management of patients who must discontinue an SGLT2 inhibitor—and, to our knowledge, provides the first real-world characterization of the clinical course after a switch to a DPP-4 inhibitor. Second, the comparison between teneligliptin and other DPP-4 inhibitors as a class was specified in the original study protocol before any analysis was performed, reducing the risk of post hoc selection bias in choosing comparator agents. Third, standardized clinical and laboratory measurements—including unattended automated office blood pressure, which is less susceptible to white-coat effects than conventional in-clinic measurement [32]—were applied uniformly across the 12-year study period using a single automated chemistry analyzer, supporting internal comparability. Fourth, the primary between-group comparison was adjusted for two pre-specified, mechanistically motivated confounders (baseline HbA1c and baseline eGFR) in a multivariable model.

Several limitations also warrant explicit consideration. (*i*) Confounding by indication is the most important threat to causal inference: as a single-center retrospective study, we cannot exclude the possibility that physicians preferentially selected teneligliptin for patients judged more likely to respond on grounds not captured in the electronic medical record (such as comorbidity profile, expected adherence, or insurance considerations), and adjustment for measured covariates cannot fully address residual confounding or channeling. Only two covariates (baseline HbA1c and eGFR) were available for adjustment; other prognostically important variables—including diabetes duration, baseline medication adherence, the presence of diabetic complications, socioeconomic status, the prescribing physician’s specialty, and the specific SGLT2 inhibitor discontinued—were not captured in the electronic medical record and could not be modeled. The marginally higher baseline HbA1c in the teneligliptin group (7.9% vs. 7.5%; *p* = 0.050) is itself a signal of such channeling and predisposes that group to a larger apparent reduction through regression to the mean. In the absence of randomization or formal causal-inference methods such as propensity-score matching or instrumental-variable analysis—which the available sample size and covariate set did not support—the −0.49% between-group difference cannot be confidently attributed to the pharmacological properties of teneligliptin. We therefore regard confounding by indication not as a peripheral caveat but as a structural limitation of the design. (*ii*) The modest sample size (*n* = 117) limits precision, particularly for secondary outcomes; consequently, the lower 95% confidence limit for the between-group HbA1c difference (−0.15%) is itself within plausible clinical range, and the upper limit (−0.83%) is correspondingly wide. The reported 80% power applies only to a simple unadjusted two-group comparison and overstates the precision available for the covariate-adjusted and exploratory subgroup analyses actually performed. (*iii*) The 3-month follow-up is insufficient to assess durability of glycemic control, cardiovascular or renal outcomes, or treatment-emergent adverse events; safety outcomes were not systematically captured. In particular, although we frame the clinical decision as a trade-off involving the cardio-renal protection conferred by SGLT2 inhibitors, no cardiovascular or renal outcome data were collected; the study therefore cannot inform that trade-off directly, and the short-term changes in body weight and blood pressure are surrogate markers only. (*iv*) Although the cohort spans 12 years, secular trends in prescribing and care may have influenced both the choice of post-switch agent and the observed outcomes. (*v*) “Other DPP-4 inhibitors” were aggregated as a single comparator to preserve statistical power, which may have obscured between-agent heterogeneity within the comparator group; we could not perform pairwise comparisons of teneligliptin against individual agents. The comparator combined gemigliptin (19.7%), sitagliptin (11.1%), and a mix of other agents (17.9%), each represented by only 10–23 patients and differing in molecular structure, binding kinetics, half-life, and excretion pathway—precisely the within-class heterogeneity the study set out to examine. Pooling these agents both masks that heterogeneity and limits the interpretability of the teneligliptin-versus-other comparison; adequately sized cohorts for each agent would be required to address the question properly. (*vi*) The study population was Korean and largely of Asian ancestry, and prescribing patterns reflect the Korean healthcare context, in which teneligliptin is widely available; the findings may not generalize directly to other populations or healthcare systems. (*vii*) Secondary outcomes were exploratory and were not corrected for multiple comparisons. (*viii*) The reason for switching was undocumented for 28.2% (33/117) of patients; this substantial proportion of missing data on a key variable was retained for transparency but was not addressed through imputation or formal sensitivity analysis. (*ix*) No measure of medication adherence (pharmacy refill records or pill counts) was available, so differential adherence between the groups cannot be excluded as a contributor to the observed HbA1c difference. (*x*) Although changes in other glucose-lowering agents were excluded by protocol, changes in non-diabetes medications that can affect glycemia—such as glucocorticoids, statins, and antihypertensive agents—were not systematically assessed or controlled. For all of these reasons, our findings should be regarded as hypothesis-generating, and confirmation in adequately powered prospective head-to-head studies is required before any change in clinical practice. Several further limitations, arising from the retrospective use of electronic medical records, deserve explicit mention. (*xi*) The cardiovascular and renal risk profile of the cohort—established atherosclerotic cardiovascular disease, heart failure, chronic kidney disease and albuminuria categories, diabetes duration, microvascular and macrovascular complications, frailty, and comorbidity burden—was not systematically recorded and could not be characterized; consequently we cannot determine whether SGLT2 inhibitors were discontinued in relatively low-risk patients or in individuals for whom organ protection was the primary indication. (*xii*) The original indication for SGLT2 inhibitor therapy could not be reliably ascertained; most patients are presumed to have been treated primarily for glycemic control, but because the study period (2013–2025) preceded the current emphasis on heart-failure, chronic-kidney-disease, and atherosclerotic-cardiovascular-disease indications, the indication was frequently undocumented. (*xiii*) Individualized HbA1c targets, baseline target attainment, hypoglycemia, and functional status were unavailable, limiting assessment of the clinical relevance of the observed HbA1c differences. (*xiv*) The urinary albumin-to-creatinine ratio was markedly skewed and substantially incomplete (available in 63 of 117 patients at baseline and in only 12 patients per group for the paired within-group analysis); although we additionally analyzed it non-parametrically, these estimates are imprecise. (*xv*) The multivariable model explained only a small proportion of the variance in HbA1c change (R^2^ = 0.18; adjusted R^2^ = 0.15), indicating that most of the between-patient variation was driven by unmeasured factors and further underscoring the exploratory nature of the adjusted between-group estimate.

## 5. Conclusions

In this single-center retrospective cohort of adults with type 2 diabetes, switching from an SGLT2 inhibitor to a DPP-4 inhibitor preserved overall glycemic control over 3 months, albeit with modest increases in body weight and blood pressure that reflect the loss of SGLT2-inhibitor-specific metabolic and hemodynamic effects. A pre-specified subgroup analysis identified a 0.49 percentage-point greater mean HbA1c reduction with teneligliptin than with the pooled group of other DPP-4 inhibitors, and this between-group difference persisted after adjustment for baseline HbA1c and eGFR. Although the limitations of an observational design require that this finding be regarded as hypothesis-generating rather than confirmatory, the difference should be interpreted cautiously, because confounding by indication and the limited covariate adjustment cannot be excluded as explanations; it is sufficient to motivate—rather than to establish—prospective comparative evaluation of whether DPP-4 inhibitors can be regarded as interchangeable in this setting. Two practical implications follow. First, when sustained SGLT2 inhibitor therapy is not feasible, clinicians should explicitly weigh the trade-off between the cardio-renal benefits of SGLT2 inhibitors and the tolerability advantages of DPP-4 inhibitors, recognizing that the choice of replacement agent—not merely the class—may influence short-term glycemic outcomes. Second, individual DPP-4 inhibitor pharmacology, including binding kinetics, excretion route, and pleiotropic effects, deserves greater consideration in routine prescribing decisions than is current practice. To advance this evidence base, an adequately powered prospective head-to-head randomized trial—ideally a pragmatic, multicenter design with at least 12 months of follow-up and pre-specified subgroup analyses by baseline renal function and prior SGLT2 inhibitor exposure—is needed to confirm whether between-agent differences within the DPP-4 inhibitor class are clinically meaningful in this specific scenario, to identify the patient subgroups most likely to benefit, and to evaluate longer-term cardiovascular, renal, and safety outcomes. In particular, the study does not establish that the replacement strategy was clinically appropriate, that cardio-renal protection was preserved, or that teneligliptin is superior to any individual alternative DPP-4 inhibitor; it demonstrates only that short-term glycemic control was broadly maintained after switching while body weight and blood pressure modestly increased. A DPP-4 inhibitor should be regarded as an unavoidable substitute when an SGLT2 inhibitor cannot be maintained rather than a therapeutically equivalent one, and these findings cannot by themselves guide changes in clinical practice.

## Figures and Tables

**Table 1:** Baseline clinical characteristics of patients switching from SGLT2 inhibitors to DPP-4 inhibitors.

	Baseline
Clinical parameters	
Age (years)	60.5 ± 11.1
Male (%)	64 (54.7%)
Bwt (kg)	67.5 ± 9.4
BMI (kg/m^2^)	25.5 ± 3.2
SBP (mmHg)	131.7 ± 19.3
DBP (mmHg)	77.3 ± 10.9
SGLT2i types	
Dapagliflozin	56 (47.9%)
Empagliflozin	54 (46.2%)
Enavogliflozin	3 (2.6%)
Ipragliflozin	4 (3.4%)
Laboratory parameters	
AST (U/L)	27.6 ± 12.9
ALT (U/L)	27.4 ± 13.6
BUN (mg/dL)	17.8 ± 6.6
Cr (mg/dL)	0.84 ± 0.41
eGFR (mL/min/1.73 m^2^)	89.3 ± 22.6
ACR (mg/g)	86.8 ± 219.0
HbA1c (mmol/mol, %)	61 ± 15 (7.7 ± 1.4%)

Data are expressed as mean ± standard deviation or number (%). ACR, albumin-to-creatinine ratio; ALT, alanine aminotransferase; AST, aspartate aminotransferase; BMI, body mass index; BUN, blood urea nitrogen; Bwt, body weight; Cr, creatinine; DBP, diastolic blood pressure; DPP-4i, dipeptidyl peptidase-4 inhibitor; eGFR, estimated glomerular filtration rate; HbA1c, glycated hemoglobin; SBP, systolic blood pressure; SGLT2i, sodium-glucose cotransporter-2 inhibitor.

**Table 2:** Reasons for switching from SGLT2 inhibitors to DPP-4 inhibitors.

Causes	*n* (%)
Poor blood glucose control	19 (16.2)
Genital mycotic infection	18 (15.4)
Weight loss	14 (12.0)
Nocturia	8 (6.8)
Urinary tract infection	6 (5.1)
Increased appetite	5 (4.3)
Thirst	4 (3.4)
Urinary frequency	3 (2.6)
Insurance issues	2 (1.7)
Fatigue	1 (0.9)
Polyuria	1 (0.9)
Urticaria	1 (0.9)
Abdominal pain	1 (0.9)
Decreased eGFR	1 (0.9)

Data are expressed as number (%). DPP-4i, dipeptidyl peptidase-4 inhibitor; eGFR, estimated glomerular filtration rate; SGLT2i, sodium-glucose cotransporter-2 inhibitor.

**Table 3:** Changes in clinical and laboratory parameters from baseline to 3 months after switching to DPP-4 inhibitors.

	Baseline	After	*p*
Clinical parameters			
Bwt (kg)	67.5 ± 9.4	68.2 ± 9.8	<0.001
BMI (kg/m^2^)	25.5 ± 3.2	25.9 ± 3.3	<0.001
SBP (mmHg)	131.7 ± 19.3	136.0 ± 16.4	0.005
DBP (mmHg)	77.3 ± 10.9	79.2 ± 12.0	0.042
Laboratory parameters			
AST (U/L)	27.6 ± 12.9	28.9 ± 13.0	0.663
ALT (U/L)	27.4 ± 13.6	29.2 ± 16.0	0.442
BUN (mg/dL)	17.8 ± 6.6	17.7 ± 7.0	0.296
Cr (mg/dL)	0.84 ± 0.41	0.87 ± 0.39	0.842
eGFR (mL/min/1.73 m^2^)	89.3 ± 22.6	86.6 ± 21.8	0.279
ACR (mg/g)	86.8 ± 219.0	127.6 ± 300.8	0.461
HbA1c (mmol/mol, %)	61 ± 15 (7.7 ± 1.4)	60 ± 16 (7.6 ± 1.5)	0.157

Data are expressed as mean ± standard deviation. *p* values were calculated using the paired *t*-test. ACR, albumin-to-creatinine ratio; ALT, alanine aminotransferase; AST, aspartate aminotransferase; BMI, body mass index; BUN, blood urea nitrogen; Bwt, body weight; Cr, creatinine; DBP, diastolic blood pressure; eGFR, estimated glomerular filtration rate; HbA1c, glycated hemoglobin; SBP, systolic blood pressure.

**Table 4:** Baseline clinical and laboratory characteristics of the teneligliptin and other DPP-4 inhibitors groups.

Parameter	Teneligliptin Group		Other DPP-4 Inhibitors Group		*p*
	*n*	Baseline	*n*	Baseline	
Clinical parameters					
Age (years)	60	59.5 ± 9.3	57	61.6 ± 12.7	0.308
Bwt (kg)	52	67.7 ± 8.9	43	67.2 ± 10.1	0.805
BMI (kg/m^2^)	52	25.6 ± 3.0	55	25.4 ± 3.4	0.775
SBP (mmHg)	59	132.3 ± 17.0	55	131.1 ± 21.8	0.754
DBP (mmHg)	59	79.2 ± 11.4	55	75.3 ± 10.1	0.057
SGLT2i types (*p* = 0.228)					
Dapagliflozin		27 (45.0%)		29 (50.9%)	
Empagliflozin		27 (45.0%)		27 (47.4%)	
Enavogliflozin		2 (3.3%)		1 (1.8%)	
Ipragliflozin		4 (6.7%)		0 (0%)	
Laboratory parameters					
AST (U/L)	56	29.3 ± 16.7	51	31.7 ± 12.4	0.304
ALT (U/L)	56	29.2 ± 13.6	51	28.3 ± 14.6	0.995
BUN (mg/dL)	55	16.1 ± 5.1	53	18.9 ± 8.4	0.061
Cr (mg/dL)	55	0.82 ± 0.31	53	0.92 ± 0.44	0.263
eGFR (mL/min/1.73 m^2^)	49	91.6 ± 20.3	40	83.5 ± 25.8	0.191
ACR (mg/g)	33	62.1 ± 107.0	30	187.8 ± 36.3	0.722
HbA1c (mmol/mol, %)	60	63 ± 14 (7.9 ± 1.3)	57	58 ± 16 (7.5 ± 1.4)	0.050

Data are expressed as mean ± standard deviation or number (%). *n* indicates the number of available observations. Between-group comparisons of baseline values were performed using the independent-samples *t*-test (continuous variables) or the chi-square/Fisher’s exact test (categorical variables). ACR, albumin-to-creatinine ratio; ALT, alanine aminotransferase; AST, aspartate aminotransferase; BMI, body mass index; BUN, blood urea nitrogen; Bwt, body weight; Cr, creatinine; DBP, diastolic blood pressure; DPP-4i, dipeptidyl peptidase-4 inhibitor; eGFR, estimated glomerular filtration rate; HbA1c, glycated hemoglobin; SBP, systolic blood pressure; SGLT2i, sodium-glucose cotransporter-2 inhibitor.

**Table 5:** Within-group changes from baseline (before) to 3 months (after) in the teneligliptin group.

Parameter	Teneligliptin Group			*p*
	*n*	Before	After	
Clinical parameters				
Bwt (kg)	43	66.9 ± 8.5	67.5 ± 9.0	0.025
BMI (kg/m^2^)	43	25.3 ± 2.8	25.5 ± 3.0	0.043
SBP (mmHg)	42	132.1 ± 17.3	134.3 ± 16.4	0.364
DBP (mmHg)	41	78.8 ± 11.8	78.9 ± 11.6	0.958
Laboratory parameters				
AST (U/L)	41	29.3 ± 16.7	28.9 ± 13.8	0.062
ALT (U/L)	41	29.2 ± 13.6	30.8 ± 16.4	0.257
BUN (mg/dL)	40	16.1 ± 5.1	17.6 ± 6.3	0.045
Cr (mg/dL)	40	0.82 ± 0.31	0.85 ± 0.34	0.497
eGFR (mL/min/1.73 m^2^)	38	91.6 ± 20.3	90.3 ± 18.7	0.106
ACR (mg/g) *	12	19.5 (9.2–52.0)	19.2 (9.5–31.3)	0.233
HbA1c (mmol/mol, %)	60	63 ± 14 (7.9 ± 1.3)	60 ± 14 (7.6 ± 1.3)	0.009

Data are expressed as mean ± standard deviation. *p* values were calculated using the paired *t*-test. * The urinary albumin-to-creatinine ratio (ACR) was markedly skewed and is reported as the median (interquartile range) and analyzed using the Wilcoxon signed-rank test. *n* indicates the number of available observations. ACR, albumin-to-creatinine ratio; ALT, alanine aminotransferase; AST, aspartate aminotransferase; BMI, body mass index; BUN, blood urea nitrogen; Bwt, body weight; Cr, creatinine; DBP, diastolic blood pressure; DPP-4i, dipeptidyl peptidase-4 inhibitor; eGFR, estimated glomerular filtration rate; HbA1c, glycated hemoglobin; SBP, systolic blood pressure; SGLT2i, sodium-glucose cotransporter-2 inhibitor.

**Table 6:** Within-group changes from baseline (before) to 3 months (after) in the other DPP-4 inhibitors group.

Parameter	Other DPP-4 Inhibitors Group			*p*
	*n*	Before	After	
Clinical parameters				
Bwt (kg)	37	67.1 ± 10.4	68.3 ± 10.6	0.006
BMI (kg/m^2^)	37	25.6 ± 3.5	26.1 ± 3.7	0.004
SBP (mmHg)	34	128.2 ± 12.0	138.2 ± 16.9	0.002
DBP (mmHg)	34	74.6 ± 10.3	79.7 ± 13.0	0.007
Laboratory parameters				
AST (U/L)	35	31.7 ± 12.4	28.5 ± 8.9	0.073
ALT (U/L)	35	28.3 ± 14.6	25.9 ± 11.6	0.279
BUN (mg/dL)	40	18.9 ± 8.4	18.2 ± 8.3	0.311
Cr (mg/dL)	39	0.91 ± 0.44	0.92 ± 0.46	0.911
eGFR (mL/min/1.73 m^2^)	34	83.5 ± 25.8	83.8 ± 25.5	0.886
ACR (mg/g) *	12	20.4 (10.7–221.3)	30.1 (14.1–186.3)	0.791
HbA1c (mmol/mol, %)	57	59 ± 15 (7.5 ± 1.4)	60 ± 18 (7.6 ± 1.7)	0.236

Data are expressed as mean ± standard deviation. *p* values were calculated using the paired *t*-test. * The urinary albumin-to-creatinine ratio (ACR) was markedly skewed and is reported as the median (interquartile range) and analyzed using the Wilcoxon signed-rank test. *n* indicates the number of available observations. ACR, albumin-to-creatinine ratio; ALT, alanine aminotransferase; AST, aspartate aminotransferase; BMI, body mass index; BUN, blood urea nitrogen; Bwt, body weight; Cr, creatinine; DBP, diastolic blood pressure; DPP-4i, dipeptidyl peptidase-4 inhibitor; eGFR, estimated glomerular filtration rate; HbA1c, glycated hemoglobin; SBP, systolic blood pressure; SGLT2i, sodium-glucose cotransporter-2 inhibitor.

**Table 7:** Between-group comparison of the change from baseline to 3 months (after − before).

Parameter	Teneligliptin Group		Other DPP-4 Inhibitors Group		*p*
	*n*	Change (95% CI)	*n*	Change (95% CI)	
Clinical parameters					
Bwt (kg)	43	0.6 (0.1 to 1.1)	37	1.2 (0.4 to 2.1)	0.217
BMI (kg/m^2^)	43	0.2 (0.0 to 0.4)	37	0.5 (0.2 to 0.8)	0.158
SBP (mmHg)	42	2.3 (−2.7 to 7.3)	34	10.1 (3.8 to 16.3)	0.050
DBP (mmHg)	41	0.1 (−2.7 to 2.9)	34	5.1 (1.5 to 8.6)	0.270
Laboratory parameters					
AST (U/L)	41	−0.4 (−4.6 to 3.8)	35	−3.2 (−6.7 to 0.3)	0.136
ALT (U/L)	41	1.6 (−3.2 to 6.4)	35	−2.3 (−6.7 to 2.0)	0.052
BUN (mg/dL)	40	1.5 (0.1 to 2.8)	40	−0.8 (−2.3 to 0.7)	0.047
Cr (mg/dL)	40	0.03 (−0.01 to 0.07)	39	−0.00 (−0.05 to 0.05)	0.009
eGFR (mL/min/1.73 m^2^)	38	−1.3 (−5.7 to 3.1)	34	0.2 (−3.1 to 3.5)	0.146
ACR (mg/g) *	12	−4.3 (−27.9 to 3.1)	12	2.5 (−10.8 to 21.9)	0.090
HbA1c (mmol/mol, %)	60	−0.36 (−0.62 to −0.09)	57	0.12 (−0.08 to 0.32)	0.005

Data are expressed as the mean change (95% confidence interval). *p* values for between-group differences in change were calculated using the independent-samples *t*-test. * The urinary albumin-to-creatinine ratio (ACR) was markedly skewed and is reported as the median change (range) and analyzed using the Mann–Whitney U test. *n* indicates the number of available observations. ACR, albumin-to-creatinine ratio; ALT, alanine aminotransferase; AST, aspartate aminotransferase; BMI, body mass index; BUN, blood urea nitrogen; Bwt, body weight; Cr, creatinine; DBP, diastolic blood pressure; DPP-4i, dipeptidyl peptidase-4 inhibitor; eGFR, estimated glomerular filtration rate; HbA1c, glycated hemoglobin; SBP, systolic blood pressure; SGLT2i, sodium-glucose cotransporter-2 inhibitor.

## Data Availability

The summary data underlying the means, standard deviations, and other measures reported in this article, including the values used to construct the figures, are contained within the article and its Supplementary Materials. The individual patient-level dataset cannot be made publicly available because it constitutes sensitive human research participant data containing potentially identifying clinical information; the Institutional Review Board of Chungnam National University Hospital approved the study on the condition that these data remain within the institution to protect participant privacy. De-identified individual patient-level data may be made available to qualified researchers for verification or reanalysis upon reasonable request, subject to approval by the Institutional Review Board and completion of a data-use agreement. Requests should be addressed to the independent Institutional Review Board of Chungnam National University Hospital, which serves as the data access committee (e-mail: irb@cnuh.co.kr); the corresponding author (B.J.K.; bonjeong@cnu.ac.kr) can assist in facilitating such requests.

## Supplementary Materials

The following supporting information can be downloaded at: https://www.mdpi.com/article/10.3390/medicina62081511/s1, Table S1: Univariate linear regression analysis of the relationship between reasons for switching and changes in HbA1c; Table S2: Multiple linear regression analysis of factors associated with HbA1c reduction.

## References

[1] Adler A.I., Coleman R.L., Leal J., Whiteley W.N., Clarke P., Stevens R.J., Holman R.R. (2024). Post-trial monitoring of a randomised controlled trial of intensive glycaemic control in type 2 diabetes extended from 10 years to 24 years (UKPDS 91). Lancet.

[2] Marso S.P., Bain S.C., Consoli A., Eliaschewitz F.G., Jódar E., Leiter L.A., Lingvay I., Rosenstock J., Seufert J., Warren M.L. (2016). Semaglutide and cardiovascular outcomes in patients with type 2 diabetes. N. Engl. J. Med..

[3] Heerspink H.J.L., Stefánsson B.V., Correa-Rotter R., Chertow G.M., Greene T., Hou F.F., Mann J.F.E., McMurray J.J.V., Lindberg M., Rossing P. (2020). Dapagliflozin in patients with chronic kidney disease. N. Engl. J. Med..

[4] Zinman B., Wanner C., Lachin J.M., Fitchett D., Bluhmki E., Hantel S., Mattheus M., Devins T., Johansen O.E., Woerle H.J. (2015). Empagliflozin, cardiovascular outcomes, and mortality in type 2 diabetes. N. Engl. J. Med..

[5] Wanner C., Inzucchi S.E., Lachin J.M., Fitchett D., von Eynatten M., Mattheus M., Johansen O.E., Woerle H.J., Broedl U.C., Zinman B. (2016). Empagliflozin and progression of kidney disease in type 2 diabetes. N. Engl. J. Med..

[6] American Diabetes Association Professional Practice Committee (2026). 9. Pharmacologic approaches to glycemic treatment: Standards of Care in Diabetes—2026. Diabetes Care.

[7] Nakamura I., Maegawa H., Tobe K., Tabuchi H., Uno S. (2021). Real-world evidence for long-term safety and effectiveness of ipragliflozin in Japanese patients with type 2 diabetes mellitus: Final results of a 3-year post-marketing surveillance study (STELLA-LONG TERM). Expert Opin. Pharmacother..

[8] Bak M., Chi S.A., Jeon K., Kim H., Cho H., Lee H.S., Park K.M. (2024). Discontinuation rates, clinical effects and provocation factors of SGLT-2 inhibitor in the real world. Sci. Rep..

[9] Mathieu C., Kozlovski P., Paldánius P.M., Foley J.E., Modgill V., Evans M., Serban C. (2017). Clinical safety and tolerability of vildagliptin—Insights from randomised trials, observational studies and post-marketing surveillance. Eur. Endocrinol..

[10] Nishimura R., Kato H., Kisanuki K., Oh A., Onishi Y., Hasegawa Y., Cai Z., Tajima Y., Aoyama N. (2019). Treatment patterns, persistence and adherence rates in patients with type 2 diabetes mellitus in Japan: A claims-based cohort study. BMJ Open.

[11] Deacon C.F. (2020). Dipeptidyl peptidase 4 inhibitors in the treatment of type 2 diabetes mellitus. Nat. Rev. Endocrinol..

[12] Göke R., Eschenbach P., Dütting E. (2015). Efficacy of vildagliptin and sitagliptin in lowering fasting plasma glucose: Results of a randomized controlled trial. Diabetes Metab..

[13] Morimoto T., Sakuma I., Sakuma M., Tanaka A., Node K., Otsuka Y., Yokoyama K., Yokota Y., Iwakura K., Kasai T. (2019). Randomized Evaluation of Anagliptin vs Sitagliptin On low-density lipoproteiN cholesterol in diabetes (REASON) trial: A 52-week, open-label, randomized clinical trial. Sci. Rep..

[14] Tang Y.Z., Wang G., Jiang Z.H., Yan T.T., Chen Y.J., Yang M., Meng L.C., Zhu Y.J., Li C.G., Li Z. (2015). Efficacy and safety of vildagliptin, sitagliptin, and linagliptin as add-on therapy in Chinese patients with T2DM inadequately controlled with dual combination of insulin and traditional oral hypoglycaemic agent. Diabetol. Metab. Syndr..

[15] Wu Y.J., Guo X., Li C.J., Li D.Q., Zhang J., Yang Y., Kong Y., Guo H., Liu D.M., Chen L.M. (2015). Dipeptidyl peptidase-4 inhibitor, vildagliptin, inhibits pancreatic beta cell apoptosis in association with its effects suppressing endoplasmic reticulum stress in db/db mice. Metabolism.

[16] Monami M., Dicembrini I., Mannucci E. (2014). Dipeptidyl peptidase-4 inhibitors and heart failure: A meta-analysis of randomized clinical trials. Nutr. Metab. Cardiovasc. Dis..

[17] Halabi A., Maatouk H., Siegler K.E., Faisst N., Lufft V., Klause N. (2013). Pharmacokinetics of teneligliptin in subjects with renal impairment. Clin. Pharmacol. Drug Dev..

[18] Kimura S., Inoguchi T., Yamasaki T., Yamato M., Ide M., Sonoda N., Hirano K., Takayanagi R. (2016). A novel DPP-4 inhibitor teneligliptin scavenges hydroxyl radicals: In vitro study evaluated by electron spin resonance spectroscopy and in vivo study using DPP-4 deficient rats. Metabolism.

[19] Korea I.Q.V.I.A. (2026). UBIST (Ubist Pharmaceutical Information System) Nationwide Pharmaceutical Market Research Database.

[20] Sagara M., Suzuki K., Aoki C., Tanaka S., Taguchi I., Inoue T., Aso Y. (2016). Impact of teneligliptin on oxidative stress and endothelial function in type 2 diabetes patients with chronic kidney disease: A case–control study. Cardiovasc. Diabetol..

[21] Craddy P., Palin H.J., Johnson K.I. (2014). Comparative effectiveness of dipeptidylpeptidase-4 inhibitors in type 2 diabetes: A systematic review and mixed treatment comparison. Diabetes Ther..

[22] Bae J.H., Han K.D., Ko S.H., Yang Y.S., Choi J.H., Choi K.M., Kwon H.S., Won K.C. (2022). Diabetes fact sheet in Korea 2021. Diabetes Metab. J..

[23] Rosenstock J., Perkovic V., Johansen O.E., Cooper M.E., Kahn S.E., Marx N., Alexander J.H., Pencina M., Toto R.D., Wanner C. (2019). Effect of linagliptin vs placebo on major cardiovascular events in adults with type 2 diabetes and high cardiovascular and renal risk: The CARMELINA randomized clinical trial. JAMA.

[24] Vilsbøll T., Krarup T., Madsbad S., Holst J.J. (2003). Both GLP-1 and GIP are insulinotropic at basal and postprandial glucose levels and contribute nearly equally to the incretin effect of a meal in healthy subjects. Regul. Pept..

[25] Kim H.J., Kim Y.S., Lee C.B., Choi M.G., Chang H.J., Kim S.K., Park J.Y., Lee B.W., Kim B.J., Kim C.H. (2021). Efficacy and safety of switching to teneligliptin in patients with type 2 diabetes inadequately controlled with dipeptidyl peptidase-4 inhibitors: 52-week results from a prospective observational study. Diabetes Ther..

[26] McGuire D.K., Alexander J.H., Johansen O.E., Perkovic V., Rosenstock J., Cooper M.E., Wanner C., Kahn S.E., Toto R.D., Zinman B. (2019). Linagliptin effects on heart failure and related outcomes in individuals with type 2 diabetes mellitus at high cardiovascular and renal risk in CARMELINA. Circulation.

[27] Pujadas G., De Nigris V., Prattichizzo F., La Sala L., Testa R., Ceriello A. (2017). The dipeptidyl peptidase-4 (DPP-4) inhibitor teneligliptin functions as antioxidant on human endothelial cells exposed to chronic hyperglycemia and metabolic high-glucose memory. Endocrine.

[28] Nabeno M., Akahoshi F., Kishida H., Miyaguchi I., Tanaka Y., Ishii S., Kadowaki T. (2013). A comparative study of the binding modes of recently launched dipeptidyl peptidase IV inhibitors in the active site. Biochem. Biophys. Res. Commun..

[29] Zhang Z., Jin X., Yang C., Li Y. (2019). Teneligliptin protects against hypoxia/reoxygenation-induced endothelial cell injury. Biomed. Pharmacother..

[30] Kim Y., Kang E.S., Jang H.C., Kim D.J., Oh T., Kim E.S., Choi K.M., Kim H.J., Park J.H., Park S.W. (2019). Teneligliptin versus sitagliptin in Korean patients with type 2 diabetes inadequately controlled with metformin and glimepiride: A randomized, double-blind, non-inferiority trial. Diabetes Obes. Metab..

[31] Kurozumi A., Okada Y., Sugai K., Tanaka Y. (2018). Comparison of the effects of teneligliptin and sitagliptin, two dipeptidyl peptidase 4 inhibitors with different half-lives, on glucose fluctuation and glucagon-like peptide-1 in type 2 diabetes mellitus. J. UOEH.

[32] Roerecke M., Kaczorowski J., Myers M.G. (2019). Comparing automated office blood pressure readings with other methods of blood pressure measurement for identifying patients with possible hypertension: A systematic review and meta-analysis. JAMA Intern. Med..

