# Supplementary material for: Glycemic Outcomes After Switching from SGLT2 Inhibitors to DPP-4 Inhibitors in Type 2 Diabetes, with a Comparison of Teneligliptin and Other Agents: A Retrospective Cohort Study
Source: Medicina (Kaunas). 2026 Aug 6;62(8):1511. doi: 10.3390/medicina62081511 (PMC13514766; doi:10.3390/medicina62081511)
Supplement: Supplementary file 1 [file medicina-62-01511-s001.zip › medicina-4430768-supplementary.pdf]

Supplementary Materials

Table S1. Univariate linear regression analysis of the relationship between reasons for switching and changes in HbA1c.

Each documented reason for switching from an SGLT2 inhibitor to a DPP-4 inhibitor was entered as an independent variable in a univariable linear regression model with the change in HbA1c from baseline to 3 months as the dependent variable.

Variable	Unstandardized β	SE	95% CI	Standardized β	*t*	*p*
Poor blood glucose control	−0.07	0.26	−0.60 to 0.45	−0.03	−0.270	0.788
Vaginitis	0.34	0.27	−0.19 to 0.87	0.14	1.290	0.201
Weight loss	−0.13	0.29	−0.71 to 0.46	−0.05	−0.424	0.673
Nocturia	0.01	0.37	−0.74 to 0.75	0.00	0.022	0.982
Urinary tract infection	−0.09	0.43	−0.94 to 0.76	−0.02	−0.214	0.831
Increased appetite	−0.06	0.46	−0.98 to 0.86	−0.01	−0.132	0.895
Thirst	−0.83	0.51	−1.84 to 0.17	−0.18	−1.648	0.103
Urinary frequency	0.50	0.59	−0.67 to 1.67	0.10	0.853	0.396
Insurance issues	−0.02	0.72	−1.45 to 1.41	−0.00	−0.025	0.980
Fatigue	0.18	1.01	−1.83 to 2.20	0.02	0.183	0.855
Polyuria	0.29	1.01	−1.72 to 2.30	0.03	0.283	0.778
Urticaria	0.08	1.01	−1.93 to 2.09	0.01	0.083	0.934
Abdominal pain	−0.73	1.01	−2.73 to 1.28	−0.08	−0.722	0.473
Decreased eGFR	−1.23	1.00	−3.22 to 0.76	−0.14	−1.233	0.221

CI, confidence interval; eGFR, estimated glomerular filtration rate; HbA1c, glycated hemoglobin; SE, standard error.

Table S2. Multiple linear regression analysis of factors associated with HbA1c reduction.

Multivariable linear regression model of the change in HbA1c from baseline to 3 months, adjusted for treatment group (teneligliptin vs. other DPP-4 inhibitors), baseline HbA1c, and baseline estimated glomerular filtration rate.

Variable	Unstandardized β	SE	95% CI	Standardized β	*t*	*p*
(Intercept)	0.60	0.71	−0.82 to 2.02	—	0.837	0.405
Teneligliptin	−0.57	0.20	−0.97 to −0.16	−0.28	−2.769	0.007
Baseline HbA1c	−0.11	0.07	−0.26 to 0.03	−0.16	−1.593	0.115
Baseline eGFR	0.01	0.00	0.00 to 0.02	0.26	2.662	0.009

Model summary: $R^2 = 0.1773$, adjusted $R^2 = 0.1483$, $F = 6.11$, $p < 0.001$. CI, confidence interval; eGFR, estimated glomerular filtration rate; HbA1c, glycated hemoglobin; SE, standard error.
